# Changes on the Surface of the SiO_2_/C Composite, Leading to the Formation of Conductive Carbon Structures with Complex Nature of DC Conductivity

**DOI:** 10.3390/ma14092158

**Published:** 2021-04-23

**Authors:** Piotr Okoczuk, Marcin Łapiński, Tadeusz Miruszewski, Piotr Kupracz, Leszek Wicikowski

**Affiliations:** 1 Faculty of Applied Physics and Mathematics, Gdańsk University of Technology, ul. Narutowicza 11/12, 80-233 Gdańsk, Poland; marcin.lapinski@pg.edu.pl (M.Ł.); tadeusz.miruszewski1@pg.edu.pl (T.M.); 2 Institute of Fluid-Flow Machinery, Polish Academy of Sciences, Fiszera 14, 80-231 Gdańsk, Poland; piokupra@pg.edu.pl

**Keywords:** sol–gel, silica/carbon composite, nanographite, conductive polymers, DC conductivity

## Abstract

Sol–gel layers have been the subject of many studies in recent decades. However, very little information exists about layers in which carbon structures are developed in situ. Using the spin-coating method, we obtained thin iron-doped SiO_2_/C composite films. The results of Raman spectroscopy showed that our samples consisted of graphitic forms and polymers. The latter’s contribution decreases with rising temperature. FTIR and EDS studies show changes in carbon distribution on top of the layer, depending on the sintering temperature. The samples sintered at 800 °C showed a significant increase in the contribution of carbon forms to the layer’s surface. Therefore, high conductivity can be observed in this sample. The results of XPS spectroscopy showed that the contribution of sp^3^ hybridized carbon increases after etching. The total electrical conductivity, studied by a DC four-wire technique, increased with the temperature and showed almost linear characteristics with significant changes below 150 K. The reduced activation energy plot has a positive temperature coefficient, which is a characteristic property of the conductive polymers in a metallic regime of conductivity.

## 1. Introduction

Carbon forms have been a very common topic of research in recent decades. Carbon-based compounds are versatile, cheap, and have structures that are easy to manipulate. In particular, their conductive properties have attracted the attention of scientists all around the world. There are many studies about the optical and electric properties of silica/carbon composites, where carbon forms are delivered ex situ [1,2,3,4,5,6]. The composites of silica and carbon are of great interest to researchers developing high-capacity anodes for lithium-ion batteries, as silicon has 10 times better potential in capacity for lithium ions than commercially produced graphite anodes [7]. Many composites of silica and carbon were developed to maximize the capacity of layers [7,8,9]. However, there is a significant lack of information in the literature regarding the electrical properties of sol–gel-prepared silica-carbon composites doped with transition metals, where carbon forms develop in situ. This approach could be a large step to obtain very cheap, simple to manufacture material, which is characterized by sufficiently high conductivity and has the potential of application in electrochemical devices.

The presence of Fe ions in the layers has a crucial role in carbon formation. Iron can be embedded in the SiO_2_ matrix or located within the pores of SiO_2_ [10]. Brasil et al. [11] showed that Fe ions can attach to acetylacetone groups and catalyze their epoxidation to other structures, mostly cyclohexene, which is a building block of graphite and other layered structures of carbon. Moreover, conductive polymers doped with FeCl_3_ can be enhanced to have metal-like properties [12,13,14,15,16,17].

In this paper, the sol–gel method was used to obtain a mixture that is homogenous on the molecular level. However, temperature treatment has an influence on composite structure development. This study shows a novel approach to in situ prepared composites, especially to surface manipulation possibilities. It may also affect electrochemical fields of study, as silica-nanographite composites are very promising materials for lithium-ion storage. An in situ approach of synthesis decreases the cost of preparation significantly. In the following chapters, we show that structural changes on top of the layer play a crucial role in the electrical properties of such composites.

## 2. Materials and Methods

### 2.1. Materials and Preparation of Layers

Acid-catalyzed hydrolysis was carried out by tetraethoxysilane (TEOS) (Alfa Aesar, purity of at least 98%, molar mass 208.33 g/mol), 0.1 M hydrochloric acid (HCl). Fe ions were delivered by the addition of FeCl_3_ (Merck-Schuchardt purity of at least 98%, molar mass 162.21 g/mol). Sucrose (SUC) (Chempur, p.a., molar mass 342.3 g/mol, sucrose content at least 99.8%) was the main source of carbon. Acetic anhydride (Ac_2_O) (Chempur, p.a., molar mass 102.09 g/mol) was added to slow down condensation. The molar proportions were 23.8:0.6:8.3:33.9:33.8 for SUC:HCl:FeCl_3_:Ac_2_O:TEOS, respectively.

The first step of preparation was dissolving SUC, FeCl_3_, and Ac_2_O in HCl. After the addition of each component, the solution was given time to homogenize. The second step was to add TEOS and wait 2 h before spin-coating the substrates. Then, a drop of the prepared sol with a volume of 0.05 mL was dropped on the spinning quartz substrate with a rotation speed of 2000 rpm. The size of the substrate was about 1.5 × 2 cm^2^. The next day, the samples were dried in air at 80 °C for an hour. The final step of preparation, carried out 4 days after the coating, was the carbonization of each sample at a different temperature (600, 800, and 1000 °C) for an hour, with a heat rate of 10 °C/min. This took place in a hydrogen atmosphere, with a flow rate of 100 mL/min.

### 2.2. Characterization

FTIR analysis in the IR region of 4000–550 cm^−1^ was carried out using the PerkinElmer Frontier spectrometer (PerkinElmer Inc., Waltham, MA, USA) in attenuated total reflection (ATR) mode. Scanning electron microscopy (SEM) images, using SE mode, were taken by an FEI Quanta FEG 250 microscope (FEI Company, Hillsboro, OR, USA), with an accelerating voltage of 20 kV. The microscope was equipped with an EDS detector, which was used for chemical composition analysis. Raman spectroscopy was carried out by an Ar laser (wavelength of 514 nm, power of 2–3 mW) using a confocal micro-Raman spectrometer InVia Renishaw (Renishaw plc, Wotton-under-Edge, UK). Low-temperature direct current (D.C.) conductivity was measured in a galvanostatic mode, with the help of Keithley 2400 precision current source (Keythley Instruments, Solon, OH, USA). The voltage response was collected with a Keysight 34970A multimeter (Keysight Technologies Inc., Santa Rosa, CA, USA), with a built-in multiplexer for data acquisition. The whole system worked with BenchLink Data Logger 3 software (Keysight Technologies Inc., Santa Rosa, CA, USA). The sample was held in a self-prepared measurement cell. Four gold electrodes were sputtered on the layer by magnetron sputtering, and silver wires were attached using a silver paste (DuPont 4922). Electrical measurements were carried out in a temperature range from liquid nitrogen (~77 K) to the freezing point of water (~273 K). The temperature was controlled by an N-type thermocouple (TERMOAPARATURA WROCŁAW, Wrocław, Poland). The XPS measurement was performed using an Argus (Omicron NanoTechnology, Taunusstein, Germany) X-ray photoelectron spectrometer. The photoelectrons were excited by a Mg-Kα X-ray source. The X-ray anode was operated at 15 keV and 300 W. The XPS measurement was performed at room temperature under ultra-high vacuum conditions, with pressures below 1.1 × 10^−8^ mbar. To obtain the depth profile of concentration, the sample was sputtered by Ar ions (FDG 150 ion source mounted in the analytic chamber) for 15 min. Data analysis was performed with the CASA XPS software package (Casa Software Ltd., ver. 2.3.23., Devon, UK), using a Shirley background subtraction and the least-square Gaussian–Lorentzian curve-fitting algorithm.

## 3. Results and Discussion

### 3.1. Raman Spectroscopy

**Figure 1:**
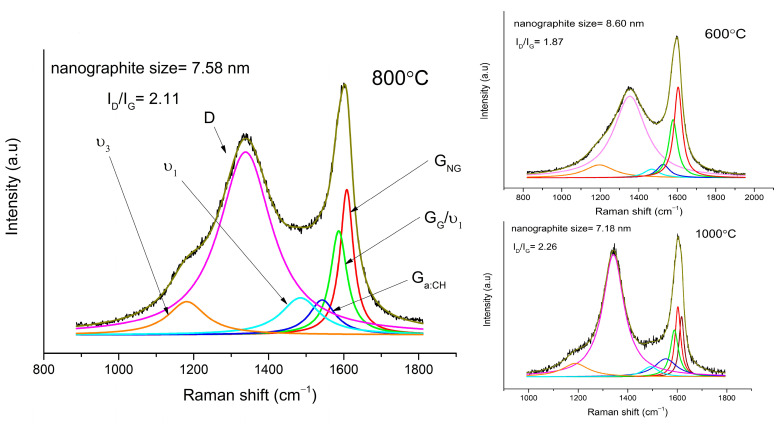
Fitted first—order Raman spectrum of sample sintered at 800 °C, 600 °C, and 1000 °C.

**Figure 2:**
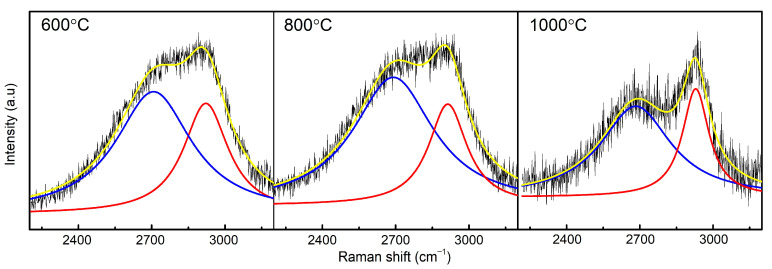
Fitted second—order Raman spectrum of samples sintered at 600, 800, and 1000 °C.

Raman spectroscopy results show three major peaks, which can be observed mainly in amorphous forms of carbon and polymers (Figure 1 and Figure 2). The D peak can be observed at 1353, 1338, and 1342 cm^−1^ for samples sintered at 600, 800, and 1000 °C, respectively. The D peak is due to the breathing mode of sp^2^ atom rings of carbon and it comes from a double resonance at the K point [18,19]. The G peak is due to the E_2g_ stretching mode. It comes from high-frequency phonon in the Γ point [18,19]. In graphite and graphene, it is usually placed near 1580 cm^−1^, but it can be shifted to 1600 cm^−1^ in graphite nanocrystalline form, and to 1520 cm^−1^ for amorphous hydrogenated carbon (a:CH) [20,21].

The G peak was decomposed to three peaks:

1. The first one is located at 1604, 1608, and 1602 cm^−1^ for samples sintered at 600, 800, and 1000 °C, respectively. It comes from nanocrystalline graphite (NG) and is upshifted in samples sintered at 600 °C and 800 °C because of the small contribution of the D’ peak. With the rising temperature, graphene planes grow mainly in the planar direction, which explains the contribution to growth of in-plane defect mode D’. Only in the sample sintered at 1000 °C was it possible to separate the D’ peak, located at 1617 cm^−1^.
2. The second peak is at 1578, 1586, and 1588 cm^−1^ for samples sintered at 600, 800, and 1000 °C, respectively. Its presence is often observed in graphite, graphene (G), and other layered structures of carbon [18,19,20].
3. The third G peak is at 1526, 1543, and 1553 cm^−1^ for samples sintered at 600, 800, and 1000 °C, respectively, and can be assigned to a:CH. The higher the temperature, the more upshifted the G peak is. This slight upshift can be a result of larger tension in bonds [22].

Two more peaks are present in our spectrum. We assigned them to ν_3_ and ν_1_ modes of polyconjugated molecules. They are usually placed at 1150–1200 cm^−1^ and 1450–1490 cm^−1^, respectively. The position of these peaks upshifts with a rising chain-length, especially the latter [23,24]. In our samples, the ν_3_ peak is at 1195, 1181, and 1188 cm^−1^, and ν_1_ is at 1467, 1484, and 1492 cm^−1^ for samples sintered at 600, 800, and 1000 °C, respectively. In poly(p-phenylenevinylene), the ν_1_ peak can even be at 1586 cm^−1^, which can also be ascribed to the second peak (G) [16].

The ratio of the D and G band intensity is equivalent to the disorder in graphite structure: if the ratio equals 1, it means that graphitic structure has no defects. It is apparent that the ratio increases with the growing temperature. The narrowing of peaks with rising temperature also provides information about the higher ordering of carbon sintered at higher temperatures [18,25]. The ratio of the integral intensity of the *D* and *G* peaks can also be used to calculate the size of nanographite grains [25,26]. Using Equation (1), the size of the nanographite grains was calculated to be 8.6, 7.5, and 7.1 nm, for samples sintered at 600, 800, and 1000 °C, respectively.

(1) $$L_{a}=\left(2.4\times 10^{-10}\right)\lambda^{4}{\left(\frac{I_{D}}{I_{G}}\right)}^{-1},$$

where *λ* is laser wavelength, and *I_D_* and *I_G_* are integral intensities of band *D* and *G*.

The second-order Raman spectra of the samples are very broad and blurred (Figure 2). The position of the main bands can only be determined in the sample sintered at 1000 °C, where the 2D band is at 2683 cm^−1^ and D + D’ is at 2928 cm^−1^. The 2683 cm^−1^ band, known as the 2D band, is known in graphene structures and is an overtone of the D peak [19]. In 1-layer graphene, this band is downshifted to 2650 cm^−1^. In the analyzed samples, the 2D band is broad and it is impossible to determine its exact position; therefore, we can assume that the samples exhibit a wide range of structure thickness [18]. At 2928 cm^−1^, we observe the D + D’ band, which is a result of a combination of the D and D’ peaks. The D + D’ band is also induced by disorder and its usual location is around 2900 cm^−1^ [19]. In the range of 2925–2970 cm^−1^, bands from C-H bond are also present [27].

### 3.2. FTIR Spectroscopy

**Figure 3:**
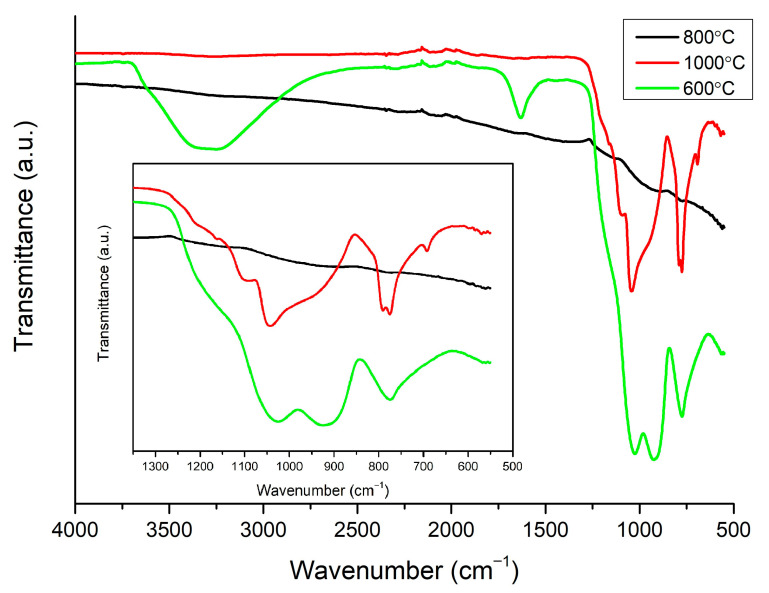
FTIR spectra of all samples. To clarity, region of 1350–550 cm^−1^ is enlarged.

**Figure 4:**
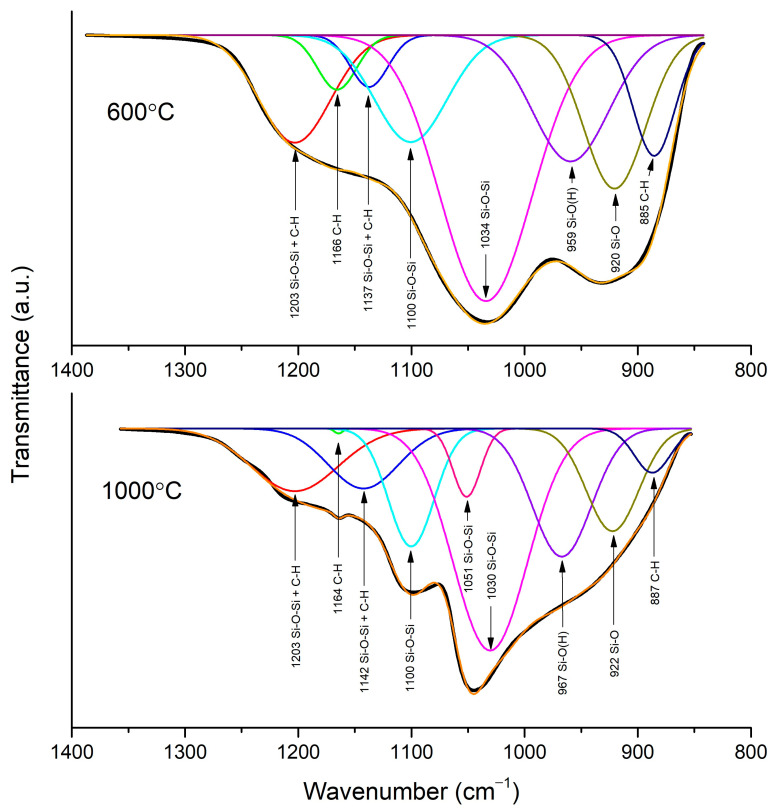
Deconvolution of region 800–1350 cm^−1^ of spectra of samples sintered at 600 °C and 1000 °C.

An FTIR analysis in ATR mode was performed to investigate the evolution of the silica network upon different temperature treatments. Only the layer sintered at 800 °C exhibits transmittance, which is characteristic for graphitic forms of carbon; no signs of silica were observed (Figure 3) [28]. The spectra of the samples sintered at 600 °C and 1000 °C show absorption bands, which is characteristic for silica materials. To investigate silica network growth, the exact position of peaks found in the spectra of samples sintered at 600 °C and 1000 °C was obtained from deconvolution (Figure 4). In the first steps of the fitting, the position of peaks had to be fixed to obtain a good quality of deconvolution. In the sample sintered at 600 °C, it can be noticed that bands at 959, 920, and 885 cm^−1^ have a big contribution to the spectrum, and they were assigned to Si-O(H) stretching vibrations, Si-O non-bridging broken bonds, and C-H bonds, respectively [29,30,31]. The intensity of the bands decreases with the growing temperature of sintering, which is a sign of clenching and developing of a SiO_2_ network (Figure 3). The drop in the intensity of the bands is also related to a change in the position of the Si-O(H) band to higher wavelengths, which were observed before for better-condensed gels [29]. The progress in condensation can also be noticed from the vanishing of a band from O-H bond vibrations, which are present only in the sample sintered at 600 °C. The band at 1034 cm^−1^ in the spectrum of the sample sintered at 600 °C and bands at 1051 and 1030 cm^−1^ in the spectrum of the sample sintered at 1000 °C correspond to Si-O-Si symmetric stretching. The band at 1100 cm^−1^, recorded for both samples, is assigned to Si-O-Si symmetric stretching in linear structures of Si-O-Si. Asymmetric stretching of this bond is also noticeable at 1137 and 1142 cm^−1^ for samples sintered at 600 and 1000 °C, respectively. The band at 1203 cm^−1^ for those samples is probably a combination of Si-O-Si asymmetric stretching in cyclic structures of SiO_2_ and C-H bond. This band, as well as the band at 1142 cm^−1^, is broad, as many forms of hydrogenated carbon are possible in amorphous structures sintered in hydrogen [30,32]. The band at 1166 and 1165 cm^−1^ for the spectra of samples sintered at 600 and 1000 °C was assigned to rocking vibrations of C-H bond in –CH_3_ groups. The band at 773 cm^−1^ in the spectrum of the sample sintered at 600 °C is assigned to Si-O stretching (Figure 3 inset). This band creates a doublet in the spectrum of the sample sintered at 1000 °C and is correlated with the band at 695 cm^−1^. Those bands are characteristic for quartz and other crystalline forms of SiO_2_ [33,34,35].

### 3.3. SEM and EDS

**Figure 5:**
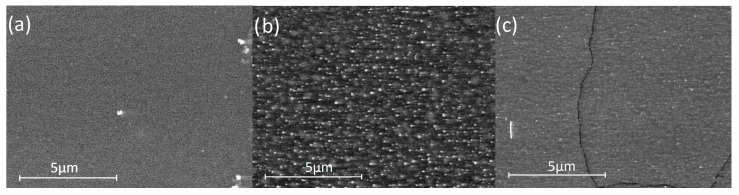
SEM images of layers sintered at (**a**) 600 °C (**b**) 800 °C (**c**) 1000 °C.

As pictured in Figure 5, the layers have a homogenous porous structure of various pore sizes. Additionally, on the basis of cross section SEM images, the thickness of the layers was measured to be about 1.5 µm for all samples. From EDS measurements (Table 1), it is apparent that the sample sintered at 800 °C is rich in carbon on top of the layer, in comparison to samples sintered at 600 °C and 1000 °C.

### 3.4. XPS Analysis

**Figure 6:**
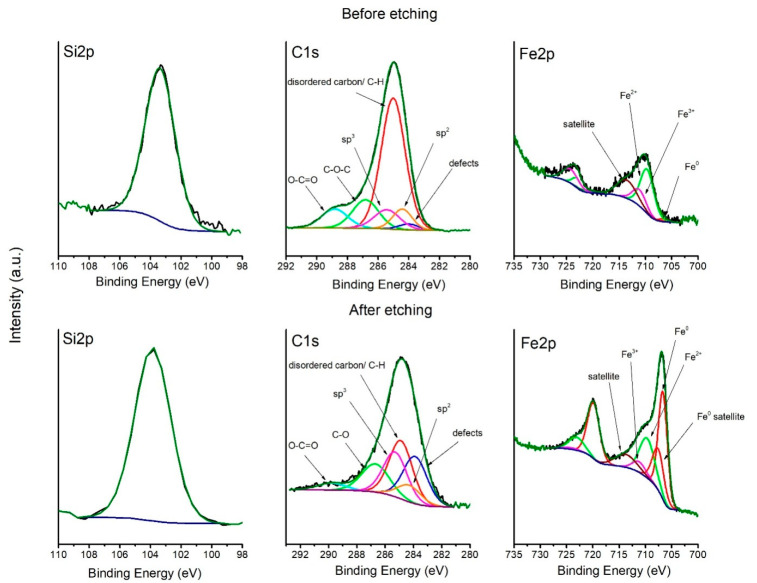
Deconvolution of Si2p, C1s, and Fe2p spectra before and after etching.

The XPS spectra of C1s, Si2p, and Fe2p regions and its deconvolutions are shown in Figure 6. The C1s peak of the non-etched sample shows a big shoulder in high bonding energy, which originates from the oxidation of the surface. Deconvolution of the C1s spectrum shows six peaks, which were ascribed to in-plane defects of carbon, sp^2^ carbon, disordered carbon and C-H bond, sp^3^ carbon, C-O bond, and O-C=O bond, respectively, at: 284.0, 284.4, 285.0, 285.4, 286.8, and 288.8 eV [36,37,38,39,40,41,42]. On the surface, the peak which comes from dangling bonds on the edge of the graphite flakes, and the C-H bond in polymers dominates, which may be a result of a highly reducing atmosphere or the presence of polymers on the surface [38,39]. After 15 min of etching by Ar^+^ ions, the contribution of this peak decreases. It can be seen that the defect carbon peak contribution increases after etching, which is a result of argon ion sputtering. The contribution of sp^3^ carbon is higher after etching, which suggests that the carbon in the pores has a tendency to transform to the sp^3^ hybridization upon the capillary forces in the clenching pores. The Si2p peak, before etching, is placed at 103.4 eV, but after etching, its position switches to 103.8 eV, which is a sign of SiO_2_ formation in the samples [43,44,45]. The Si2p peak at a lower binding energy is also related to the downshift of O1s peak (not shown). It was seen before that the presence of transition metal oxide lowers the position of both the Si2p and the O1s peak [45]. Deconvolution of Fe2p before etching is characteristic for oxides of iron in the Fe^2+^ and Fe^3+^ state at 709.9 and 711.6 eV, respectively. Only a small contribution of Fe^0^ can be seen before etching at 706.8 eV. After etching, a metallic form of iron (Fe^0^) dominates [46,47,48].

### 3.5. Electric Measurements

**Figure 7:**
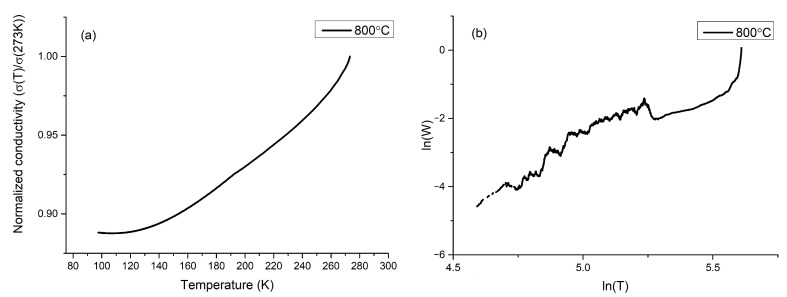
(**a**) Normalized conductivity of sample sintered at 800 °C (**b**) reduced activation energy plot of sample sintered at 800 °C.

The electrical properties of the analyzed materials were investigated via a conventional D.C. four-wire method. The measurements were made for all samples. It was noticed that samples sintered at 600 and 1000 °C showed a conductivity of ~4 × 10^−6^ (Ω·cm)^−1^, which was beyond the scope of our measuring devices. Samples sintered at 800 °C showed conductivity on the level of 45–50 (Ω·cm)^−1^ with low activation energy for the conduction mechanism. At a lower temperature regime, the total electrical conductivity decreases with the temperature, which is a characteristic behavior in the case of semiconducting materials. It must be underlined that the conductivity does not obey the Arrhenius equation. Many models were applied in order to describe the conductivity mechanism in our sample: a small and large polaron hopping, variable range hopping, the Lee and Ramakrishnan model, and models for conductive polymers [49,50,51,52]. However, none of these models gave satisfying results, which may indicate that a model which is not described in the literature may be predominant in our materials. Normalized conductivity versus temperature is shown in Figure 7a. On the plot, it can be seen that conductivity has an almost linear character between 150 and 250 K, with a small disturbance at 193 K. The conductivity graph shows a plateau below 150 K. Similar behavior was observed in graphite-polymer composites and conductive polymers [12,53,54,55,56]. The electronic regime of conductivity of polymers can be deduced from the slope of reduced activation energy, which can be obtained by the logarithmic derivative *W*:

(2) $$W=-\frac{\partial ln\rho \left(T\right)}{\partial \ln\left(T\right)},$$

where *W* is the reduced activation energy, ρ is the resistivity of the sample, and *T* is the temperature. If the reduced activation energy has a positive slope, the polymer is in the metallic regime. If the reduced activation energy has a negative slope, the polymer is in the insulating regime. If reduced activation energy is independent of temperature, it is in the critical regime, which is a transition between a metallic and an insulating regime [12]. As can be seen in Figure 7b, reduced activation energy shows a positive slope, which describes a metallic regime of conductivity, which may correspond to conductance through a polymer or polymer-graphite composite. It cannot be ignored that the estimated temperatures of inflection points of conductivity plot are in agreement with a temperature of γ relaxation of short segments of CH_2_ and β relaxation [57,58].

## 4. Conclusions

The Raman study has shown that in our samples, layered carbon coexists with carbon polymers. Graphitic planes are growing with the rising temperature of sintering, as well as the ratio of graphitic to polymeric forms. FTIR studies have shown that silica in our samples has a porous structure, which clenches with the rising temperature of sintering. FTIR study has also shown a significant change on the surface of the sample sintered at 800 °C, as only graphitic forms are present in the spectrum of that sample. SEM and EDS studies confirm that the surface of the samples sintered at 800 °C is different from the others, which can be related to a bigger carbon contribution on the surface, in contrast to the other samples. XPS studies confirm the existence of polymers, sp^2^, and sp^3^ forms of carbon. XPS analysis of the etched sample has also shown that more sp^3^ carbon forms are present in the deeper parts of the sample, which confirms that silica pores clench during the sintering. The observed structural changes on top of the layer suggest that in the sample sintered at 800 °C, carbon was pushed out from the SiO_2_ pores and the carbon was pyrolyzed enough to create connected graphite planes, while in the 1000 °C sample, the carbon was locked and clenched in SiO_2_ pores. In the 600 °C sample, the graphitic forms were not condensed enough to form conductive paths (as established from water bands observed in FTIR studies).

The conductivity of the sample sintered at 800 °C changes slightly between 45 and 50 (Ω·cm)^−1^, depending on the temperature. This conductivity value was seen in the FeCl_3_-doped PPV [59] and composites of carbon fibers, or carbon black with polymers. Knowledge about amorphous structure development upon temperature treatment [60] suggests that we may obtain layered structured carbon bridged by polymers. Electrical studies imply that the conductivity of the sample sintered at 800 °C has a complex character and cannot be explained by well-known models. The positive slope of the reduced activation energy and changes in the conductivity at temperatures of polymer relaxation indicate the participation of the polymers in the electric transport. It is possible that in our sample, two carrier transport mechanisms occur simultaneously: one through graphite and another one through polymers, which connect the graphite grains.

## Figures and Tables

**Table 1:** Results of EDS of samples sintered at 600, 800, and 1000 °C.

	Atomic Percent (±1%)		
Element	600 °C	800 °C	1000 °C
C	19%	43%	0%
O	54%	34%	61%
Si	25%	22%	36%
Fe	3%	2%	3%

## Data Availability

The data presented in this study are openly available at: https://mostwiedzy.pl/pl/piotr-okoczuk,1038332-1/research-data.
